# Mapping the Global Landscape of Dengue Fever Research: A Scientometric Analysis

**DOI:** 10.1155/ipid/8275781

**Published:** 2025-11-24

**Authors:** Meisam Dastani, Jalal Mardaneh, Reza Ahmadi

**Affiliations:** ^1^Infectious Diseases Research Center, Gonabad University of Medical Sciences, Gonabad, Iran; ^2^Department of Microbiology, School of Medicine, Infectious Diseases Research Center, Gonabad University of Medical Sciences, Gonabad, Iran; ^3^School of Medicine, Infectious Diseases Research Center, Gonabad University of Medical Sciences, Gonabad, Iran

**Keywords:** dengue fever, publications, scientometric, Scopus

## Abstract

**Background:**

Dengue fever (DF), an illness caused by the dengue virus and mainly spread by *Aedes* mosquitoes, has become a global health concern.

**Objective:**

With the considerable increase in the incidence of DF in recent decades, there is a need for a comprehensive scientometric analysis to map the landscape of scientific research in this field.

**Methods:**

This descriptive study utilized scientometric techniques for scientific analysis. The population consisted of all scientific publications on DF indexed in the Scopus database. Data were analyzed using Biblioshiny, a web-based graphical interface for the Bibliometrix package in the R programming language.

**Results:**

The study identified 3172 publications on DF from the Scopus database, with a significant growth observed since the early 1990s and peak publication years in 2019 (*n* = 202) and 2020 (*n* = 203). India, China, and the United States emerged as the most productive countries, forming the core of the international research collaboration network. Keyword co-occurrence analysis revealed dominant research focuses on “dengue virus,” “*Aedes aegypti*,” and “vector control.” Thematic mapping showed that foundational themes such as “dengue fever” and “*Aedes aegypti*” are central but underdeveloped, while niche themes like “rainfall” and “temperature” are highly developed but less central. Thematic evolution analysis showed a clear shift from virology-centered topics (pre-2005) to predictive modeling, climate impact, and machine learning applications (2020–2024).

**Conclusion:**

The comprehensive scientometric analysis shows that scientific production in the field of DF has significantly increased in recent decades. International collaborations play a vital role in advancing research and combating DF. Thematic analysis and mapping of research topics highlight significant advancements and the need for further research in specific areas. Continued collaborations and innovations are essential for effectively addressing the challenges of DF.

## 1. Introduction

Dengue viruses (DENVs) belong to the dengue complex within the genus *Flavivirus* and the family Flaviviridae. There are four antigenically related but distinct serotypes of DENV: DENV-1, DENV-2, DENV-3, and DENV-4. These serotypes can cause a range of illnesses, from asymptomatic dengue infection to dengue fever (DF), dengue hemorrhagic fever (DHF), and dengue shock syndrome (DSS) [1, 2]. Despite concerted efforts to control the disease, the incidence of DF has increased dramatically over the past few decades, with outbreaks occurring sporadically across various regions [3, 4]. The complexity of DF's epidemiology is further compounded by factors such as climate change, global warming, and the lack of public awareness regarding preventive measures [5].

Interestingly, recent studies have highlighted unique aspects of the disease, such as the confirmation of DENV RNA in ocular tissues following panophthalmitis [6] and the importation and spread of DENV serotypes across continents, contributing to the complexity of its global epidemiology [7]. Therefore, the increasing global burden of DF, its diverse clinical presentations, and the varying epidemiological patterns necessitate a scientometric analysis to map the landscape of scientific research on this infectious disease.

Scientometrics, a branch of the broader field of bibliometrics, involves the quantitative analysis of scientific literature to measure and analyze scientific productivity, research impact, and the dissemination of knowledge. This approach is particularly valuable in understanding research trends, identifying key areas of focus, and uncovering gaps in the existing literature [8, 9]. By examining publication patterns, citation networks, and collaboration dynamics, scientometrics provides insights into how research on specific topics, such as all types of diseases, evolves over time [10, 11]. This method helps policymakers, researchers, and public health professionals to make informed decisions based on comprehensive data about scientific activities.

In the context of infectious diseases, scientometric analyses have been employed to assess research output and impact across various diseases. For instance, studies have been conducted on the scientometric profiles of diseases like HIV/AIDS [12], tuberculosis [13], brucellosis [14], and malaria [15]. These analyses typically focus on metrics such as the number of publications, citation counts, authorship patterns, and the geographical distribution of research efforts. They also highlight influential researchers, institutions, and countries contributing to the field. Such studies provide a clear picture of the research landscape, highlighting both prolific areas of investigation and under-researched topics that require more attention.

In the field of DF, several studies have been conducted with a scientometric approach, each of which has addressed a part of the global or regional situation in this field. In this regard, Zyoud [16], analyzing 19,581 scientific documents published until 2015, demonstrated that the United States, India, Brazil, Thailand, and France were the leading countries in the production of science related to DF. Similarly, Dwivedi [17] also reported an upward growth in dengue-related scientific publications between 1989 and 2015, emphasizing that countries, such as Vietnam and Taiwan, despite their smaller areas, rank higher than some other countries, including India, in terms of research quality and consistency.

At the regional level, Maula et al. [18] examined the trend of DF research in Indonesia and Southeast Asia and found that despite Indonesia's relatively low share of total publications (5.9%), the annual growth rate of research in this country was the highest in the region (28.87%). They also found that topics, such as insect vectors, disease outbreaks, dengue viruses, and dengue vaccines, have been growing in recent years.

On the other hand, extensive analyses, including Cheong et al. [19], which reviewed nearly a century of research on the main vector of the disease, *Aedes aegypti*, demonstrated that the main thematic clusters of these studies were “using Wolbachia,” “Dengue Zika,” “worldwide diversity,” “community support,” “larvicidal activity,” “mosquito genotype-dependent,” and “sterile insect technique.” Furthermore, Liu et al. [20] reviewed studies from 1995 to 2023, highlighting the role of innovation in the areas of antiviral drugs, vaccines, and biotechnologies, such as Wolbachia-infected mosquitoes, and indicated that interdisciplinary and international collaborations have played an important role in advancing dengue research. They reported that despite significant progress, some knowledge gaps remain in topics such as virus–host interactions, the application of artificial intelligence in epidemic prediction, and the development of targeted interventions for vector control.

According to the results of previous studies, it has been observed that these studies have mainly focused on limited periods, countries, or specific topics. In addition, a comprehensive scientometric study of global research in the field of DF and the mapping and trend of thematic changes was not conducted. Therefore, the present study, using the scientometric method and the analysis of thematic clusters and collaboration networks, presented an obvious picture of the published research in the field of DF. Accordingly, the main research question was, “What have been the trends, patterns, and thematic structures in dengue fever research at the global level?” Moreover, the present work examined three key axes: (1) Which countries, authors, and institutions have had the most participation in dengue fever research? (2) What are the leading and emerging thematic clusters in this field? (3) How has the change in the structure of the topics of this scientific field been formed?

Accordingly, the objectives of the study are defined as follows:

The general objective of the present research was to conduct a comprehensive and systematic scientometric analysis of scientific publications related to DF globally.

Furthermore, the specific objectives include the following:

1. Identification of the growth trend and temporal changes in scientific publications related to DF
2. Investigation of the geographical distribution of scientific production and scientific collaborations between countries and institutions
3. Analysis of keywords and mapping the leading and emerging thematic clusters in this field
4. Explanation of the evolution of research topics in different periods

## 2. Methods

This descriptive and retrospective study employs bibliometric techniques to analyze the global scientific landscape related to DF research. The statistical population consists of all scientific publications about DF indexed in the Scopus database.

The selection of the Scopus database for retrieving bibliometric data in this research is due to its comprehensiveness, extensive coverage, and high-quality data. Scopus, as one of the largest citation and abstract databases, covers research articles from various scientific fields worldwide. The continuous updating of data allows access to the most recent research and citations. Scopus contains over 76 million records, including articles, conference papers, and book chapters from more than 5000 international publishers. This database is updated daily to ensure that the latest scientific information is available to researchers [21].

One of the prominent features of Scopus is its advanced analytical tools, which enable researchers to more accurately and comprehensively examine research trends, scientific collaboration networks, and the impact of articles. These tools include a variety of metrics, such as impact indices, co-citation analysis, and thematic analyses that assist in a more precise and comprehensive analysis of scientometric data. Utilizing these tools can provide more reliable and valid results, thereby enhancing the accuracy and credibility of the research findings [22].

Therefore, the use of the Scopus database in this research not only increases the accuracy and validity of the results but also leads to a more comprehensive analysis of bibliometric data.

To ensure transparency and reproducibility, the data for this study were collected on June 25, 2024, using the following search strategy. It should be noted that the keywords were extracted from the MeSH database, ensuring consistency with medical and scientific terminologies.

### 2.1. Search Strategy

(TITLE (“Dengue Fever”) OR TITLE (“Classical Dengue”) OR TITLE (“Classical Dengues”) OR TITLE (“Break-Bone Fever”) OR TITLE (“Break-Bone Fever”) OR TITLE (“Break-Bone Fever”).

For data analysis, the Biblioshiny tool, a web-based graphical interface for the Bibliometrix package in the R programming language, was used. Bibliometrix is a tool for visualizing information in bibliometric analyses based on scientific outputs and publications in metrics such as countries/regions, journals, authors, articles, author keywords, and research hotspots. This tool has the capability to map various scientific landscapes [23, 24].

In the present study, a set of scientometric and network analysis techniques, including co-authorship analysis, co-occurrence analysis, clustering analysis, and thematic mapping, were used to describe and analyze the structure of knowledge generated around DF. In this regard, for better visualization and interpretation of the data, several maps were drawn, including an international scientific collaboration map to identify active countries and collaborations between them, a keyword co-occurrence analysis map to reveal the salient areas and thematic orientation of the articles, and a thematic map to classify the topics into four main clusters, including niche, motor, emerging or declining, and basic themes. Finally, by drawing a thematic evolution diagram, the evolution of concepts and structural changes in the field of DF research over different periods was identified.

**Figure 1:**
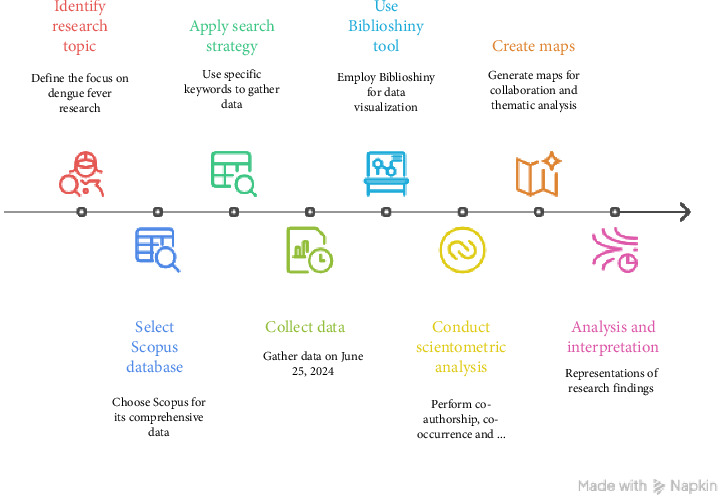
Schematic of the stages of study methodology.

The following figure presents the research methodology process schematically:

∗ The AI-assisted tool named Napkin AI (napkin.ai) was used to generate Figure 1.

## 3. Results

**Figure 2:**
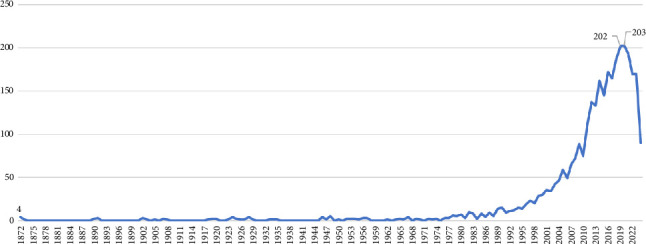
Annual trend of scientific publications on dengue fever.

A total of 3172 scientific publications related to DF were extracted from the Scopus database.  Figure 2 shows the annual trend of scientific publications in this field in the Scopus database.

The data in Figure 2 indicate that scientific publications related to DF have been indexed in the Scopus citation database since 1872. The growth of scientific publications in this field began in the early 1990s, with the highest number of publications in 2020 (203) and 2019 (202).

**Figure 3:**
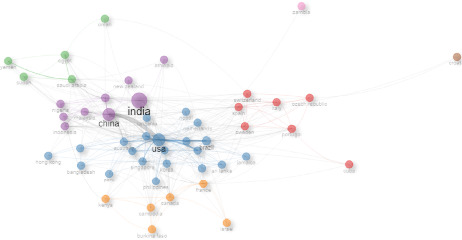
Map of scientific collaboration between countries participating in dengue fever scientific publications.

Figure 3 depicts the scientific collaboration network among countries involved in DF research. This visualization map highlights the global effort to understand and combat DF by showcasing the cooperative relationships between different nations. Each node on the map represents a country, and the size of the node correlates with the volume of scientific output from that country in the field of DF research. The lines connecting the nodes indicate collaborative research activities between countries. Prominent countries with larger nodes, such as India, China, and the United States, are seen as central players in this network, reflecting their substantial contributions and extensive partnerships in dengue research.

The data of Figure 3 indicated India, positioned centrally and depicted with a large node, is a major contributor and collaborator in DF research. Its strong connections with countries like the United States, China, and Australia highlight its role in facilitating global research partnerships. Similarly, China and the United States, also depicted with large nodes, show extensive collaboration networks, underscoring their significant involvement in the global research community. The United States, in particular, shows strong links with European countries such as the United Kingdom, France, and Germany, indicating robust transatlantic research collaborations.

The map also reveals regional clusters of collaboration, particularly among Southeast Asian countries like Malaysia, Indonesia, and the Philippines, which are heavily affected by DF. These countries have developed strong collaborative ties to address their shared challenges related to the disease. For instance, Malaysia's connections with Singapore and Indonesia highlight regional efforts to pool resources and expertise. Additionally, countries like Brazil and Mexico in Latin America exhibit strong collaborative ties, reflecting regional cooperation in addressing the dengue burden.

European countries such as Switzerland, Italy, and Spain form another cluster of collaboration, often linked with the United States, indicating a blend of intra-regional and inter-regional research partnerships. African countries, though less central, show emerging collaborations, particularly between countries like Nigeria, Tanzania, and Kenya, signaling growing participation in global dengue research efforts.

**Figure 4:**
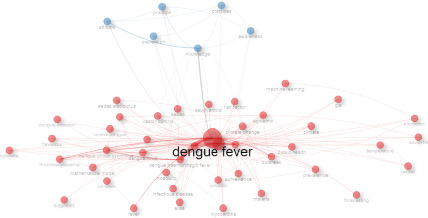
Co-occurrence map of dengue fever scientific production keywords.

The provided co-occurrence network map in Figure 4 from a scientometric analysis of DF research highlights the central themes and relationships among frequently occurring terms within the literature. At the core of this network is the term “dengue fever,” indicating its primary focus within the research. Surrounding this central node are clusters of terms representing different aspects of DF research.

The data of Figure 4 indicated red nodes signify medical and biological terms such as “dengue infection,” “severe dengue,” “dengue shock syndrome,” “*Flavivirus*,” “cytokine,” “thrombocytopenia,” “diagnosis,” and “children.” These terms are closely related and suggest a strong focus on the disease's pathology, symptoms, and medical response. Additionally, terms like “*Aedes albopictus*” and “mosquito” highlight the vector responsible for spreading DF, while “Saudi Arabia,” “Pakistan,” “climate change,” “temperature,” “rainfall,” “public health,” “outbreak,” “surveillance,” “prevalence,” “epidemic,” “risk factor,” and “vector control” emphasize the geographical, environmental, and epidemiological aspects of the disease. The presence of terms like “machine learning,” “GIS,” “prediction,” “forecasting,” and “mathematical model” suggests the use of advanced technological and computational methods to predict, track, and analyze the spread of DF.

In contrast, the blue nodes represent behavioral and awareness studies, with terms such as “attitude,” “knowledge,” “practice,” “practices,” “prevention,” and “awareness.” These nodes indicate research focused on public awareness, education, and behavioral studies related to DF prevention and control.

**Figure 5:**
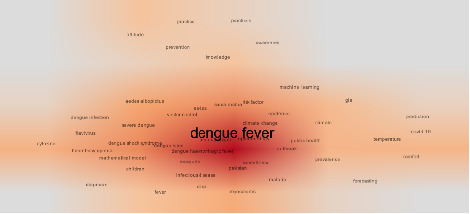
Scientific word density map in dengue fever scientific publications.

Figure 5 is a word cloud density map from a scientometric analysis of DF research. This word density map visually represents the frequency and importance of keywords associated with research on DF. Central and darker-colored words indicate topics that are more frequently addressed and hold greater significance in the scientific literature, while lighter-colored words on the periphery represent less frequently discussed topics.

At the center of the map in Figure 5, the term “dengue fever” appears larger and darker, highlighting its centrality and importance in the research field. Surrounding this central term are related keywords such as “*Aedes aegypti*,” “dengue virus,” “dengue hemorrhagic fever,” and “vector control,” indicating widespread attention to these aspects in scientific studies. Additionally, terms like “climate change,” “public health,” and “outbreak” suggest a strong focus on the connections between DF and environmental as well as public health issues.

On the periphery of the map, terms like “machine learning,” “forecasting,” “COVID-19,” and “temperature” appear, indicating emerging or specialized topics within DF research. These terms, although less prominent, highlight interactions between different subjects and potential future research directions. The word density map effectively illustrates that research on DF is moving toward a comprehensive approach, considering multiple factors related to the disease, which can aid in improving strategies for its control and prevention.

**Figure 6:**
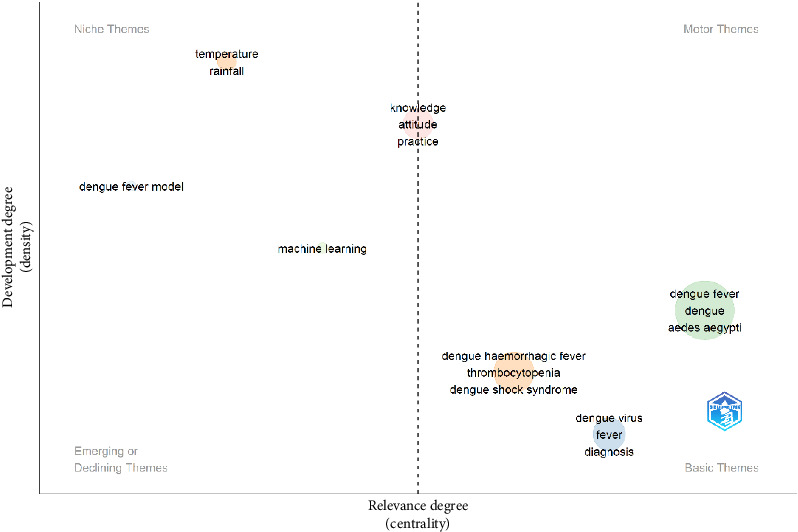
Thematic map of dengue fever scientific publications.

Figure 6 presents a thematic map of DF scientific publications. Figure 5 is divided into four main sections, each of which represents different characteristics of themes and research topics in the field of DF. These sections include foundational themes, niche themes, emerging or declining themes, and driving themes.

According to the data in Figure 6, niche themes in DF scientific publications include the topics of temperature and rainfall. These themes are considered to be specialized and niche themes, which are specialized topics that have high development but low central importance. Topics related to DF modeling are also included in this category.

The motor themes section of the diagram is empty, indicating that this study did not identify any theme that is both well-developed and has high central importance.

The placement of knowledge, attitude, and practice topics in the middle section (between motor themes and niche themes) indicates that in DF research, these topics are both well-developed and have moderate central importance. This placement suggests the topics have received adequate attention in dengue research and that research on these topics has been conducted in a balanced manner. They are not yet considered to be highly central and driving (motor themes) or completely specialized (niche themes) topics.

Emerging or declining themes include topics related to machine learning. This theme is categorized as an emerging or declining theme, indicating that it has low development and low central importance. This suggests that this topic may be new to research or is experiencing a decline in attention.

Basic themes also include topics related to DF, dengue, and *Aedes aegypti*. These themes are classified as foundational themes, which have high central importance but low development. They represent fundamental and essential topics in the field of DF. Topics related to DHF, thrombocytopenia, and DSS are also included in this section. Additionally, topics related to the keywords DENV, fever, and diagnosis are considered foundational themes.

**Figure 7:**
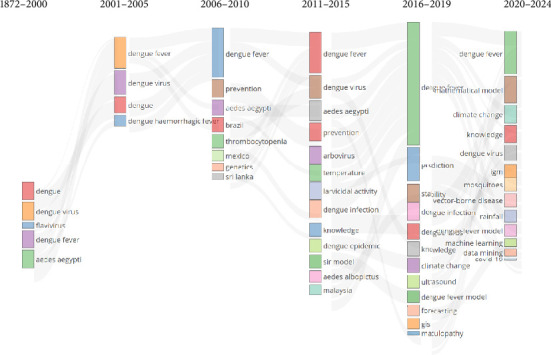
Thematic evolution of dengue fever scientific publications.

Figure 7 presents the thematic evolution of DF research from 1872 to 2024, divided into time periods of 1872–2000, 2001–2005, 2006–2010, 2011–2015, 2016–2019, and 2020–2024. Each time period displays the key topics and keywords associated with research during that period, illustrating how the focus of DF research has changed over time.

According to the data in Figure 7, the key topics during the 1872–2000 time period included the keywords: dengue, dengue virus, *Flavivirus*, and dengue fever. This indicates that the focus of research during this period was on the fundamental characteristics of the disease and its causative virus.

During the 2001–2005 time period, the key topics included the keywords: dengue, dengue virus, *Flavivirus*, dengue fever. This suggests that research during this period continued to focus on DF and its virus, but with an increased emphasis on the more severe form of the disease, DHF.

In the 2006–2010 time period, the key topics included: dengue fever, prevention, *Aedes aegypti*, Brazil, thrombocytopenia, Mexico, genetics, and Sri Lanka. This indicates that research during this period became more diverse, encompassing preventive measures and the role of the *A. aegypti* mosquito as the primary vector of the disease. Specific geographic studies (e.g., Brazil, Mexico, Sri Lanka) and topics such as genetics and thrombocytopenia were also prominent during this timeframe.

During the 2011–2015 time period, the key topics included dengue fever, dengue virus, *Aedes aegypti*, prevention, arbovirus, temperature, larvicidal activity, dengue infection, knowledge, dengue epidemic, and SIR model, *Aedes albopictus*, Malaysia. This suggests that research during this period placed a greater emphasis on other vectors such as *A. albopictus* and environmental factors like temperature. Topics related to dengue epidemiology, such as epidemics and prevention, remained important, and specific interventions such as larvicidal activities and mathematical modeling (SIR model) also gained attention.

During the 2016–2019 time period, the key topics included dengue fever, mathematical model, climate change, knowledge, dengue virus, prediction, IgM, mosquitoes, stability, vector-borne disease, dengue infection, rainfall, dengue fever model, machine learning, knowledge, data mining, COVID-19, ultrasound, dengue fever model, forecasting, GIS, and maculopathy. This indicates that during this period, the use of advanced analytical tools such as machine learning and data mining for prediction and modeling increased significantly. The impact of climate change emerged as an important topic, along with new issues like the interaction between dengue and COVID-19. The use of geographic information systems (GIS) and other modern technologies for disease outbreak prediction and understanding became more prevalent.

Similarly, during the 2020–2024 time period, the key topics have included dengue fever, mathematical model, climate change, knowledge, dengue virus, prediction, IgM, mosquitoes, stability, vector-borne disease, dengue infection, rainfall, dengue fever model, machine learning, knowledge, data mining, COVID-19, ultrasound, dengue fever model, forecasting, GIS, and maculopathy. This indicates that the final period continues many of the topics from 2016 to 2019, with an even greater emphasis on integrating machine learning and data mining into dengue research. The role of climate change remains prominent, highlighting its ongoing impact on vector-borne diseases. The topics also suggest a continued focus on mathematical modeling, prediction, and the use of advanced technologies to understand and predict dengue outbreaks.

## 4. Discussion

The data presented in the study show that scientific publications related to DF have been indexed in the Scopus database since 1872. However, it was not until the early 1990s that a significant increase in research output in this field began to emerge. This growth trajectory peaked in 2019 and 2020, with 202 and 203 publications, respectively, highlighting the intensified focus on DF research during these years. In this regard, past studies have also indicated a gradual increase in scientific output in the field of DF [20, 25]. The surge in publications in recent decades can be attributed to the increasing global burden of DF, necessitating more comprehensive and interdisciplinary research efforts.

Based on the findings of past studies, DF worldwide has increased from 557.15 per 100,000 people in 1990 to 740.4 per 100,000 in 2019 [26]. Also, the global incidence of DF increased by 1.70% annually from 1990 to 2011 and then decreased by 0.41% annually from 2011 to 2019 [27]. Therefore, based on the increase in the incidence of the disease in recent years, global research has also been increasing. Another significant finding is the importance of international cooperation in DF research, as illustrated by the scientific collaboration network map. This map underscores the pivotal roles of countries such as India, China, and the United States in fostering global partnerships. Their central positions in the network demonstrate their leadership in driving collaborative efforts, which are crucial for pooling resources, sharing expertise, and advancing knowledge in this field. In this regard, previous studies have reported an increase in DF in South Asia, especially in India, Nepal, Bangladesh, and Pakistan, in recent years [28, 29]. Also, in other Asian countries such as Indonesia, Thailand, Malaysia, the Philippines, and Vietnam, the incidence of dengue has increased in the last 3 decades [30]. In the United States, DF is also hyperendemic and has a high prevalence, and the incidence of dengue and DHFs has increased significantly [31]. These trends show the importance of continuous surveillance and the development of targeted prevention strategies to combat the increasing burden of DF worldwide, which is why the efforts of researchers from countries that are more involved in this disease in participating in scientific production are greater.

The thematic map also highlights key areas within DF research. Topics such as “dengue fever,” “*Aedes aegypti*,” and “diagnosis” are identified as highly important but require further development. In contrast, topics like “temperature” and “rainfall” are well-developed but hold less central importance in the current research landscape. Additionally, “machine learning” is recognized as an emerging or potentially declining theme, suggesting its recent introduction into the field or a shift in research focus.

DENV transmission dynamics cannot be explained by mosquito (*Aedes* vector) density alone. A complex network of variables is involved in the process (such as DENV virus virulence, heterogeneity in human immunity, vectorial capacity, and abiotic variables [climate change]). There is no available vaccine for dengue, and prevention and control of the disease have been focused on the elimination of *Aedes* mosquito populations [32, 33]. The present data showed that topics such as DF, *Aedes* vectors, detection, and prevention are recognized as very important topics, but they need further development. Fewer studies have been done in the mentioned fields, and there are many unknown aspects in these subject fields. There is a need for more extensive research and the allocation of more research funds in the field of virus virulence factors, DF pathogenesis, vectors, diagnosis, and vaccines. In contrast, data revealed topics such as climate are well-studied but less important in the current research landscape.

According to the thematic map and emerging trends in dengue research, Liu et al. reported on the widespread application of new technologies, such as artificial intelligence and machine learning, in the analysis and prediction of dengue outbreaks. The study also highlights the progress made in the development of vaccines, including Dengvaxia, Qdenga, and TV003, and the use of new vector control methods, involving the use of Wolbachia-infected mosquitoes and gene editing technologies [20]. Furthermore, Ho et al. indicated that dengue research from 1991 to 2014 was mainly focused on tropical medicine, virology, infectious diseases, parasitology, and immunology [34]. In this regard, a study conducted by Maula et al., focusing on the 10-year trend of dengue research in Indonesia and Southeast Asian countries, reported that emerging topics like disease prevalence, DENV, and vaccines are becoming the main research areas in this region [18].

Effective policies extracted from research will be key to preparing for and managing changes in the geographic range and incidence of the disease. These include implementation of vaccination campaigns in target geographical regions; improved surveillance systems; evidence-based vector control; increased people awareness of the disease; development of accurate early warning systems based on climate changes and environmental data and other factors to allow timely preventive measures to be implemented; and increased support for research to better understand the current and likely future distributions of DENV and vector (*Aedes*) [33]. Past studies have also shown that DF, *A. aegypti*, and detection are very important but need more focus. Conversely, “temperature” and “precipitation” are well-studied but less central to current research trends [35–37].

Regarding the emergence of related topics of machine learning models in DF, it can be said that these models can be used for predictive analysis of dengue outbreaks [38]. Machine learning models are used to predict the prevalence of dengue by establishing relationships between climatic factors and the increase in dengue-positive cases. These models are used to predict dengue outbreaks by analyzing meteorological data and positive data of dengue patients. Machine learning models play an important role in analyzing complex data sets and identifying patterns that can help predict the spread of DF and help with early intervention and control strategies [39, 40].

The thematic evolution of dengue research, as depicted in the thematic map, reveals a dynamic shift from initial studies focusing on the virus and disease characteristics to more complex, multidisciplinary approaches. These modern approaches encompass environmental factors, advanced modeling, and emerging global challenges. This evolution highlights the increasing complexity and sophistication of research tools and topics over time. The shift toward integrating various disciplines indicates a holistic approach to understanding and addressing the multifaceted nature of DF.

### 4.1. Limitations and Future Aspects

Although this study provides a comprehensive scientometric analysis in the field of DF, it relies only on articles indexed in Scopus and may not include some relevant studies from other sources. Also, the nature of scientometric methods is descriptive, limiting the ability to assess the quality and content of studies. In the future, combining data from multiple scientific abstract and citation databases, applying advanced text mining and network analysis methods, and focusing on emerging interdisciplinary areas such as genomics and vaccine development could help to better understand research trends to combat DF.

## 5. Conclusion

The comprehensive scientometric analysis of DF research demonstrates a significant increase in scientific output over the past few decades, driven by the growing global impact of the disease. The international collaboration network highlights the importance of global partnerships in advancing research and combating DF. The thematic evolution and mapping of research topics provide a nuanced understanding of the field's progression, indicating a move toward more complex and multidisciplinary approaches. These findings underscore the necessity for continued collaboration and innovation to effectively address the challenges posed by DF and improve global health outcomes.

## Data Availability

The data that support the findings of this study are available from the corresponding author upon reasonable request.
